# Parliament and the Profession

**Published:** 1918-08-03

**Authors:** 


### PARLIAMENT AND THE PROFESSION.

### Hospitals at the Front.

Colonel Faber asked the Under-Secretary of State for War if he will consider the advisability of having the hospitals at the Fronts some distance from fighting units, so that no mistake can possibly arise in enemy bombing operations; whether to assist this object light railways could be laid down by which to remove patients on to the main lines; and whether he will insist on the Red Cross being plainly painted on our marquees. Mr. Macpherson said that the measures suggested have already been put in hand, so far as labour is available. Hospital establishments are clearly marked.

### Hospital Ships.

Dr. Macnamara informed Sir H. Nield that the question of the best means to be adopted to secure immunity for hospital ships is under consideration, and he regretted it is not possible to make any statement on the subject at the present time.

